# 4216 A TL1 team approach to investigate attention and learning at the intracranial network level and assess the effect different cognitive rehabilitation strategies have on measures of attention and learning

**DOI:** 10.1017/cts.2020.104

**Published:** 2020-07-29

**Authors:** Sarah E. Long, Catherine Tocci, Giridhar Kalamangalam, William Perlstein, Ayse Gunduz

**Affiliations:** 1 University Of Florida Clinical and Translational Science Institute

## Abstract

OBJECTIVES/GOALS: 1) Investigate the network level interactions of attention and learning during an attention network task (ANT) and an implicit learning contextual cueing (CC) task. 2) Assess the effect attention rehabilitation strategies have on *behavioral and neural responses pre/post-attentional intervention*. METHODS/STUDY POPULATION: This study involves refractory epilepsy patients with implanted intracranial electrodes and moderate-to-severe traumatic brain injury (m/sTBI) survivors. In epileptic patients, we will identify connectivity of cortical regions via the ANT, which probes components of attention (alerting, orienting, and executive control) and a CC task that probes implicit learning. We hypothesize that modulation of attention and learning can be seen at the network level. In TBI we will assess improvement following two attention rehabilitation paradigms behaviorally; and use our results from epileptic patients to guide measurement of treatment-related neuroplastic change via scalp electroencephalography. RESULTS/ANTICIPATED RESULTS: When the proposed objectives are complete, we expect to determine how the implicit learning rate in m/sTBI changes as a result of both direct attention and metacognitive-strategy training, and discern the neuroanatomical networks associated with attention and implicit learning based on connectivity results. We expect to identify intracranial regions and networks that exhibit modulatory effects associated with attention and implicit learning. Additionally, we anticipate that deficits in attention will be mitigated following training and hypothesize that implicit learning rate will improve in TBI patients as a result of both attentional rehabilitation paradigms. DISCUSSION/SIGNIFICANCE OF IMPACT: Characterizing intracranial activity in epilepsy patients will give electrophysiology data unattainable in TBI patients. This intracranial perspective will enable us to propose mechanisms of action that may result from our interventions and enable critique of current rehabilitation treatments.

